# Evaluation of multi-environment adaptability of flour quality traits in spring wheat varieties and screening of high-quality genotypes using AMMI and GGE models

**DOI:** 10.3389/fpls.2026.1874719

**Published:** 2026-07-10

**Authors:** Na Sun, Qiannan Huang, Hui Yang, Zhihui Zhang, Wei Wang, Xuehui Zhang, Lin Ma, Dengsilamu Tuerxunbai, Hui Zou, Jialiken Madiyaer, Hongwei Geng

**Affiliations:** 1 College of Agronomy/High Quality Special Wheat Crop Engineering Technology Research Center, Xinjiang Agricultural University, Urumqi, China; 2 Yili Kazakh Autonomous Prefecture Institute of Agricultural Science/Key Laboratory of Crop Breeding and Quality Testing of Yili Prefecture, Yining, China; 3 Department of Computer Science and Information Engineering, Anyang Institute of Technology, Anyang, China

**Keywords:** AMMI model, GGE model, screening of high-quality varieties, spring wheat, wheat quality traits

## Abstract

To clarify the regulatory effects of genotype (G), natural environment (E, defined as site × year), and genotype-by-environment interaction (G × E) on the core quality traits of spring wheat and to screen high-quality, stable cultivars and rational water and nitrogen management strategies for the Ili River Valley in Xinjiang, we conducted field experiments across three typical ecological sites over two consecutive growing years. Eight spring wheat varieties belonging to four quality types (strong gluten, medium-strong gluten, medium gluten, and weak gluten) were tested under nine water–nitrogen combined management treatments (three irrigation levels × three nitrogen levels). Six core processing quality traits, including water absorption, dough stability time, and grain protein content, were measured. In accordance with revised statistical criteria, phenotypic data from all water and nitrogen management treatments were averaged for each natural environment to eliminate artificial management interference. The AMMI model, GGE biplot analysis, and multivariate comprehensive evaluation were adopted to systematically dissect trait variation, genotypic stability, and environmental adaptability. The results showed that the six quality traits were synergistically regulated by genotype, natural environment, and G × E interaction with distinct trait-specific responses. Dough extensograph area and extension resistance exhibited strong genetic dominance, with genotypic variation dominating phenotypic variation. In contrast, grain protein content was more sensitive to natural environmental fluctuation, showing relatively higher environmental dependency. Spatially, the Gongliu site presented the most favorable ecological conditions and superior comprehensive wheat quality performance among the three experimental environments. Appropriate water and nitrogen management significantly optimized spring wheat quality performance, and the optimal water–nitrogen combination for the coordinated improvement of multiple quality traits was determined. Comprehensive evaluation based on revised AMMI and GGE models demonstrated that Neimai 17 possessed the best comprehensive quality performance and wide environmental adaptability, while Hechun 137 and Xinchun 37 also exhibited stable and excellent quality characteristics across diverse natural environments. This study systematically clarifies the G × E regulatory patterns of spring wheat quality traits in arid ecological conditions, establishes an effective multi-model fusion screening system for high-quality and stable wheat cultivars, and provides a theoretical basis and technical support for germplasm improvement, regional variety layout, and green and high-efficiency production of specialized spring wheat in the Ili River Valley and similar northwest arid regions of China.

## Introduction

Wheat is one of the three major cereal crops globally, cultivated on over 220 million hectares annually and with a total production of approximately 780 million tons (Wang et al., 2025). It serves as a primary source of dietary energy and protein for approximately one-third of the world’s population. In China, wheat ranks second in grain production after rice, with an annual consumption exceeding 130 million tons. Stable wheat production is critical to national food security and socioeconomic stability (Tiwari et al., 2025). With the shift in consumer preferences from food sufficiency to quality-oriented diets, the demand for specialized and functional wheat raw materials in the food processing industry has steadily increased (Wang et al., 2024). Accordingly, modern wheat breeding objectives have shifted from yield-oriented single improvement to multi-trait coordinated optimization, comprehensively improving grain quality, yield potential, resource use efficiency, and cultivation sustainability. Processing quality is the core embodiment of the market value of wheat. Six core traits, including grain protein content, wet gluten content, dough water absorption, stability time, resistance to extension, and extensibility, directly determine the processing adaptability of wheat in terminal products such as bread, noodles, and steamed buns (Martínez-Peña et al., 2023; Kumari et al., 2024). For high-quality strong-gluten wheat, grain protein content above 14.00%, wet gluten content above 32.00%, and dough stability time longer than 10 min are essential to satisfy the dough elasticity and gas retention requirements for bread making. In contrast, weak-gluten wheat requires a grain protein content below 12.00% and a short dough stability time to meet the crisp-texture characteristic of biscuits and pastries. The precise regulation of wheat processing quality has become a key technical bottleneck restricting the high-quality development of the modern wheat industry.

However, the phenotypic expression of these key quality traits is not determined merely by genetic background but also by the complex synergistic effects of G, E, and G × E. Numerous studies have demonstrated that the quality of the same wheat variety varies significantly across different ecological regions or with interannual climatic fluctuations (Mohammadi et al., 2025; Sharma et al., 2025; Srivastava et al., 2025). Such quality instability severely limits the large-scale standardized production, targeted processing, and brand construction of specialized high-quality wheat (Li and Tao, 2022; Mirosavljevic et al., 2024).

The Ili River Valley in Xinjiang is an important spring-wheat-producing area in China with unique ecological advantages. The annual average sunshine hours exceed 2800 h, and the day and night temperature difference reaches 15 °C to 20°C, which is conducive to the accumulation of photosynthetic products and protein synthesis and provides natural conditions for the production of high-quality strong-gluten wheat. However, the ecological conditions in this region are complex and diverse. Zhaosu County, with an altitude of 1,800 to 2,000 m, has a cool climate and a short growth period. Gongliu County, with an altitude of 700 to 1,000 m, has a warm climate and superior irrigation conditions. Tekes County, with an altitude of 1,200 to 1,500 m, has a complex terrain and significant differences in soil fertility. At the same time, the region is facing increasingly tight water resource constraints, with agricultural water mainly relying on the Ili River and its tributaries and facing prominent seasonal water shortage problems. These complex ecological and resource conditions increase the environmental sensitivity of spring wheat quality traits and raise the difficulty of targeted variety selection and precise cultivation management (Zhang et al., 2022; Lin et al., 2025).

In recent years, multivariate statistical models such as the additive main effects and multiplicative interaction model and the genotype main effects and genotype-by-environment interaction biplot have become mainstream tools for analyzing crop genotype-by-environment interaction and screening stable and high-quality varieties. The AMMI model (Abakemal et al., 2016; Shanthi et al., 2025; Sood et al., 2025) decomposes the genotype-by-environment interaction effect through principal component analysis which can accurately quantify the variety stability and has been widely used in wheat quality research (da Silva et al., 2021; Liu et al., 2022; Wodebo et al., 2023; Burato et al., 2025)—for example, Kumari et al. (2024) analyzed the variation patterns of quality, phenological, and yield traits based on multi-environment experiments of wheat core germplasm resources using the AMMI model, providing an important basis for the excavation of high-quality genes. In other crops, the AMMI model has also shown strong analytical capabilities. Abdelrahman et al. (2022) quantified the genotype-by-environment interaction of rice quality traits through the AMMI model, clarifying the stability performance of different varieties (Martínez-Peña et al., 2023). Dang et al. (2024) analyzed the stability of sugar beet yield and quality using the AMMI model, providing a scientific basis for variety screening. The GGE biplot visualizes the genotype and genotype-by-environment interaction effects through singular value decomposition, which is convenient for identifying mega-environments and adapted varieties. Martínez-Peña et al. (2023) identified the mega-environments and adapted varieties of durum wheat in the Castile and León regions of Spain through the GGE biplot, providing support for regional layout. Yan et al. (2000) clarified the adapted varieties in different ecological regions through the GGE biplot in maize research, thereby promoting the regional planting of maize varieties. Gupta et al. (2023) comprehensively used selection index AMMI model and GGE biplot analysis to successfully identify the wheat genotype RAJ3765 with both high heat tolerance and good yield performance.

Nevertheless, existing studies on G × E regulation of spring wheat quality have obvious limitations. Most previous studies only focused on a small number of conventional quality indicators while ignoring critical processing-related traits such as dough water absorption and extensograph area. Moreover, most relevant studies adopted a single statistical model independently, failing to integrate the quantitative advantage of the AMMI model in variation decomposition and the visual decision-making advantage of the GGE biplot in ecological adaptation evaluation (Tunc et al., 2025). Most studies stay at the level of statistical description, lacking in-depth discussion of the physiological and ecological driving mechanism of genotype-by-environment interaction, especially the unclear influence path of water and nitrogen regulation on the interaction mode of quality traits in arid and semi-arid areas, leading to the disconnection between variety screening results and field cultivation measures.

Based on this and relying on field experiments conducted from 2022 to 2023 at three typical ecological sites in the Ili River Valley, this study set three nitrogen application levels and three irrigation levels and systematically determined six core processing quality traits of eight spring wheat varieties. By integrating the advantages of the AMMI model and GGE biplot, the study aimed to (1) clarify the characteristics of genotype environment and genotype-by-environment interaction effects on the core quality traits of spring wheat and determine the variation contribution rate of each factor, (2) screen varieties with both high quality and high stability under multi-environment conditions and clarify the adaptation law between varieties and environments, and (3) reveal the influence mechanism of water and nitrogen coupling regulation on the genotype-by-environment interaction mode of quality traits and determine the optimal water and nitrogen combination. The results of this study will fill the gap in the research on the multi-dimensional quality trait genotype-by-environment interaction of spring wheat in arid and semi-arid areas and provide theoretical support and practical reference for the breeding improvement and regional layout of high-quality and special-purpose wheat in the northwest arid areas of China.

## Materials and methods

### Experimental materials

A total of eight wheat varieties were used in the experiment, all of which are the main promoted varieties or excellent lines in the northwest spring wheat region covering different gluten strength types and genetic backgrounds. The selection criteria were as follows: adaptation to the spring sowing ecological conditions of the Ili River Valley, a wide local planting area or prominent quality potential, and coverage of different processing uses such as strong gluten, medium gluten, and weak gluten, which can fully reflect the quality diversity of wheat varieties in this region. All variety seeds were sourced from formal scientific research institutions or regional seed breeding bases, with a seed purity of no less than 98.00% and a germination rate of no less than 85.00% meeting the national wheat seed quality standards. The detailed information of each variety is shown in **Supplementary Table 1**.

### Experimental design and field management

#### Experimental sites and environmental characteristics

**Figure 1:**
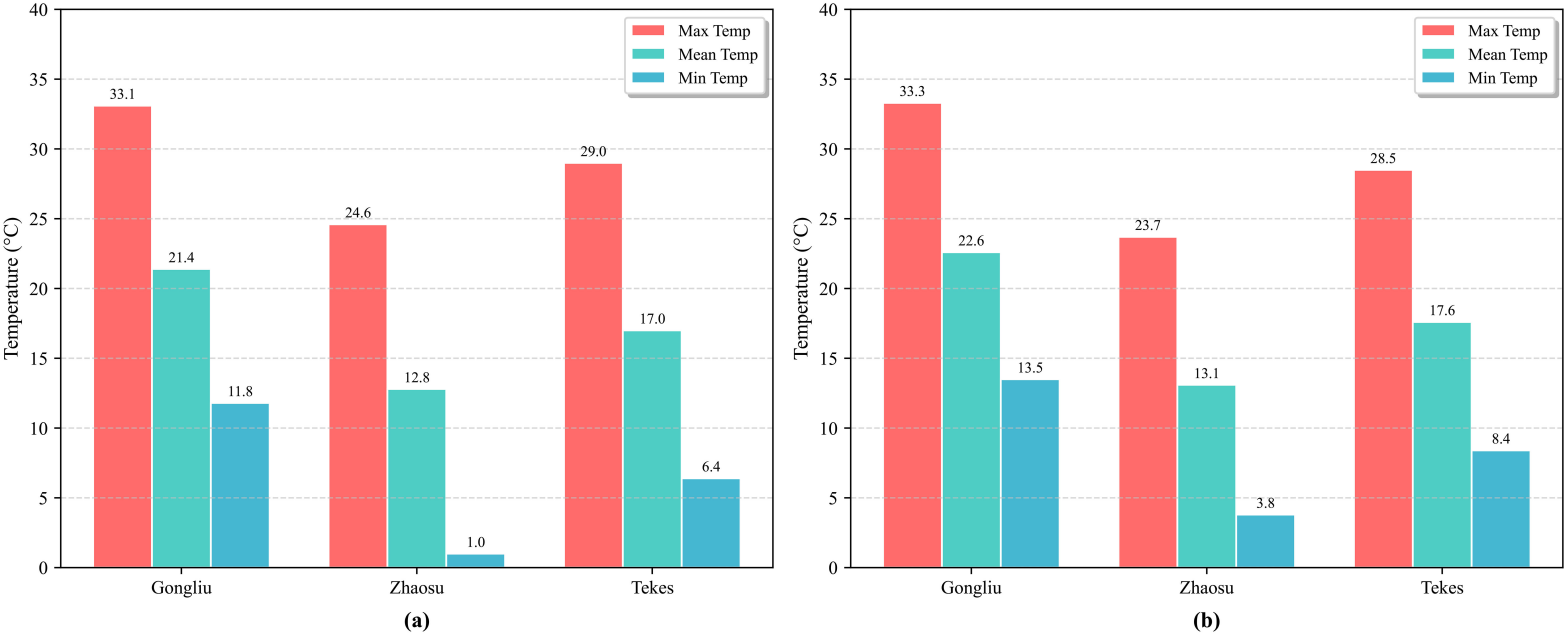
Temperature characteristics during the grain-filling period of spring wheat at three experimental sites in 2022 **(a)** and 2023 **(b)**. Bars represent the maximum temperature (red), mean temperature (green), and minimum temperature (blue) during the grain-filling stage at each site. Gongliu showed the highest mean temperature, while Zhaosu had the lowest maximum and minimum temperatures. Tekes exhibited the largest diurnal temperature range in both years.

The experiment was conducted from 2022 to 2023 in three typical ecological regions of the Ili River Valley in Xinjiang, namely, Gongliu, Zhaosu, and Tekes. All three locations are major spring-wheat-producing areas in Northwest China with distinct ecological conditions, which can effectively capture genotype-by-environment interactions. The maximum, mean, and minimum temperatures during the wheat-grain-filling period across the 2 years are presented in **Figure 1**. Gongliu (43.32° N, 82.27° E, 700–800 m a.s.l.) has irrigation-silted soil with organic matter content of 12.50–14.20 g/kg and pH 7.80–8.20. Annual precipitation ranges from 450 to 550 mm, with convenient irrigation conditions. During the grain-filling period in 2022 and 2023, the maximum temperature ranged from 33.1 °C to 33.3°C, the mean temperature was 21.4 °C–22.6 °C, and the minimum temperature was 11.8 °C–13.5°C. This site had the highest mean filling temperature among the three locations. Zhaosu (43.15° N, 81.03° E, 1,800–2,000 m a.s.l.) has chernozem soil with organic matter content of 16.80–18.50 g/kg and pH 7.50–7.90. Annual precipitation ranges from 500 to 600 mm, with a cool, humid climate and a long growing period. During grain filling, the maximum temperature was 24.6 °C–23.7°C, the mean temperature was 12.8 °C–13.1°C, and the minimum temperature was 1.0 °C–3.8 °C. This site showed the lowest temperature conditions during grain development. Tekes (43.23° N, 81.49° E, 1,200–1,400 m a.s.l.) has chestnut soil with organic matter content of 13.60–15.30 g/kg and pH 7.70–8.10. Annual precipitation ranges from 400 to 500 mm, with abundant sunshine. During grain filling, the maximum temperature was 29.0 °C–28.5°C, the mean temperature was 17.0 °C–17.6°C, and the minimum temperature was 6.4 °C–8.4°C. Notably, Tekes exhibited the largest diurnal temperature range among the three sites in both years, with differences between maximum and minimum temperatures exceeding 20°C.

#### Experimental treatment design

A split-plot experimental design was adopted, with water and nitrogen combinations as the main plots, wheat varieties as the subplots, and three replications for each treatment. Nine water–nitrogen combinations were set in the main plots, consisting of three nitrogen application levels and three irrigation levels.

The nitrogen treatments (N) included three levels, namely: N0 (no nitrogen application, control), N240 (3,600 kg/ha), and N300 (4,500 kg/ha). Urea containing 46% nitrogen was used as the nitrogen fertilizer. Of the total nitrogen, 60% was applied as basal fertilizer before sowing, and the remaining 40% was top-dressed at the jointing stage. The selected fertilizer types included urea with 46.00% nitrogen content, calcium superphosphate with 12.00% phosphorus content, potassium sulfate with 50.00% potassium content, calcium superphosphate with 180 kg per hectare of P_2_O_5_, and potassium sulfate with 75 kg per hectare of K_2_O, which were all applied as base fertilizer at one time. Nitrogen fertilizer was applied through drip irrigation in equal amounts for multiple times. Water treatments (W) consisted of three irrigation levels, namely: W300 (4,500 m³/ha), W450 (6,750 m³/ha), and W600 (9,000 m³/ha). All plots were irrigated via drip irrigation. The total water was distributed at a ratio of 3:4:3 across the reviving, jointing, and grain filling stages, respectively.

Subplot treatments included eight tested wheat varieties. A randomized block design was used in the experiment with three replications. Each plot had a row length of 5 m, a width of 4 m, and an area of 20 m^2^, with a row spacing of 0.20 m and a 1-meterm passage between plots. The seeding rate was uniformly 6 million grains per hectare. Two protective rows were set around to prevent water and nutrient flow.

#### Planting and field management

The wheat sowing time in 2022 and 2023 was from March to April. The sowing dates at the Gongliu test site were March 15, 2022 and March 12, 2023, with harvest on June 30, 2022 and June 28, 2023. The sowing dates at the Tekes test site were April 5, 2022 and April 6, 2023, with harvest on July 15, 2022 and July 18, 2023. The sowing dates at the Zhaosu test site were April 12, 2022 and April 15, 2023, with harvest on August 13, 2022 and August 16, 2023. Except for water and nitrogen treatments other management measures were uniformly implemented with reference to the local high-yield spring-wheat field standards. Base fertilizer, including diammonium phosphate at 2,250 kg per hectare and potassium chloride at 1,350 kg per hectare, was applied combined with soil preparation before sowing. Weed control adopted a combination of manual weeding and chemical weeding, with isoproturon wettable powder sprayed after sowing and before seedling emergence. Disease and pest control focused on wheat rust and aphids with high-efficiency and low-toxicity pesticides such as triadimefon and imidacloprid sprayed at a unified time.

### Sample harvest and processing

After wheat maturity, the middle three rows with an area of 6 m^2^ in each variety plot were harvested separately. After manual threshing, impurities were removed, and the grains were naturally air-dried to a moisture content of approximately 13.00%, and the plot yield was recorded by weighing. A 1-kg grain sample of each variety was taken, sealed, and stored in a refrigerator at 4°C for subsequent flour preparation and quality trait determination.

### Data collection

All quality traits were determined with three biological replicates; for each combination of variety, environment, and treatment, three representative samples were randomly selected and measured independently, and the mean value was used as the final result after eliminating abnormal values via Grubbs’ test (*P* < 0.05).

### Water absorption (%)

Water absorption was determined according to the national standard GB/T 14614-2019, Inspection of grain and oils. The rheological properties of wheat flour dough were determined using a farinograph.

### Stability time (min)

Dough stability time was measured following GB/T 14614-2019, as specified above.

### Protein content on dry basis (%)

The protein content (dry basis) was quantified using the Kjeldahl method in accordance with GB/T 5511-2008, Cereals and pulses: Determination of nitrogen content and calculation of crude protein content. Nitrogen content was multiplied by a conversion factor of 5.7 to determine the crude protein content.

### Wet gluten content (%)

Wet gluten content was assessed according to GB/T 5506.1-2008, wheat and wheat flour—Gluten content—Part 1: Determination of wet gluten by hand-washing method.

### Extensibility area (cm²)

The extensibility area was measured using an extensograph following GB/T 14615-2019, Inspection of grain and oils: Determination of rheological properties of wheat flour dough using an extensograph.

### Maximum resistance to extension (EU)

Maximum resistance to extension was evaluated under the same conditions as described for extensibility area, in compliance with GB/T 14615-2019.

### Quality types

The material quality types were classified in accordance with the National Crop Variety Approval Standards in 2017 shown in **Supplementary Table 2**. Wheat varieties were classified into four categories according to gluten strength, namely: strong gluten, medium–strong gluten, medium gluten, and weak gluten. The classification followed the principle of grading down if any index fails to meet the standard, that is, wheat with all quality indicators meeting the strong gluten standard was classified as strong gluten wheat. If any index failed to meet the strong gluten requirement but met the medium–strong gluten standard, it was classified as medium–strong-gluten wheat. If any index failed to meet the medium–strong-gluten requirement but met the medium gluten standard or even if it failed to meet the weak gluten standard, it was classified as medium-gluten wheat.

### Data analysis

#### Data preprocessing

Microsoft Excel 2021 was used to input and sort the data of six quality traits from 2022 to 2023. Missing and obvious erroneous values were removed. Grubbs’ test (*P* < 0.05) was applied to detect outliers, which were replaced with supplementary measurements. Data from 2022 and 2023 were analyzed separately before being combined, and year effects were explicitly included in subsequent analyses.

#### Descriptive statistical analysis

Data were analyzed using R software (version 4.3.1). The dplyr package was employed to calculate the mean, standard deviation (SD), and coefficient of variation (CV = SD/mean × 100%) for six quality traits to evaluate the overall variation characteristics. Data were grouped by natural environment (site × year), nitrogen level (N0, N240, and N300), and water level (W300, W450, and W600). Group means for each trait were computed, and Duncan’s multiple range test (*P* < 0.05) was conducted to assess the effects of different environments and water–nitrogen treatments on quality traits. Pearson correlation coefficients were calculated to determine the relationships among the six quality traits and to identify patterns of coordinated variation. Multi-way ANOVA was performed with site, year, genotype, nitrogen, and irrigation as fixed factors to evaluate the significance of main effects and interactions.

#### AMMI model analysis

To systematically analyze the influence mechanism of genotype, environment, and their interaction on wheat quality traits and quantify the stability performance of each genotype under multi-environment conditions, the additive main effects and multiplicative interaction model was used in this study (Anuradha et al., 2022). The model combines the additive structure of traditional analysis of variance with the dimensionality reduction ability of principal component analysis, which can effectively reveal the complex nonlinear genotype-by-environment interaction mode. The basic mathematical expression of the AMMI model is as follows:


$$Y_{ge}=\mu +\alpha_{g}+\beta_{e}+\sum_{m=1}^{t}\lambda_{m}\gamma_{gm}\delta_{em}+\epsilon_{ge}$$

*Y_ge_* represents the observed mean value of a certain quality trait such as water absorption and protein content of the *g*-th wheat genotype in the *e*-th natural environment (site × year).

*u* is the grand mean of all observed values that is the average trait value of all genotypes in all environments.

*α_g_* is the main effect of the *g*-th genotype, reflecting the deviation degree of the average performance of the variety in all environments relative to the grand mean. This effect satisfies the constraint condition $\sum_{g=1}^{G}\alpha_{g}=0$ where *G* is the total number of genotypes (which is eight in this study).

$\beta_{e}$ is the main effect of the *e*-th environment reflecting the deviation degree of the overall influence of the environment on the trait relative to the grand mean. Natural environment (E) was strictly defined as site × year (three sites × 2 years = six natural environments); water and nitrogen treatments were analyzed as independent management factors.

$\lambda_{m}$ is the singular value corresponding to the *m*-th IPCA axis whose size reflects the interaction information carried by the principal component.

$\gamma_{gm}$ is the score of the *g*-th genotype on the IPCAm.

$\delta_{em}$ is the score of the *e*-th environment on the IPCAm.

$\epsilon_{ge}$ is the residual term.

After model fitting to comprehensively evaluate the stability of each genotype the AMMI stability value was used as the core index in this study. ASV was calculated based on the scores of the first two significant IPCA axes and its formula is as follows:


$$ASV=\sqrt{{\left(\frac{SS_{PCA1}}{SS_{PCA2}}*PCA1_{score}\right)}^{2}+PCA2_{{score}^{2}}}$$

Among them, SS_PCA_*_m_* is the sum of squares of the *m*-th interaction principal component axis and PCA*m*_score_ is the score of the variety on the *m*-th IPCA axis. A smaller ASV value indicates higher stability of the genotype.

This study required that the cumulative interpretation rate of the AMMI model, that is, the proportion of interaction explained by the main effect and significant IPCA axes, should not be less than 80% to ensure that the model has good goodness of fit and explanatory power. All AMMI analyses were performed on the six natural environments.

#### GGE model analysis

The GGEbiplotGUI package of R software was used to construct the GGE model through singular value decomposition based on genotype main effects plus genotype-by-environment interaction effects (Yan et al., 2000). GGE analysis was performed on the six natural environments. The first two principal components were extracted with a cumulative interpretation rate of not less than 70.00% as the validity judgment standard. The comprehensive GGE score of each variety was calculated. The score comprehensively considered the average performance of the variety characterized by principal component 1 and stability characterized by the comprehensive vector length of principal component 1 and principal component 2. Its calculation formula is as follows:


$$GGE=\frac{PC1_{\mathrm{var}}*PC1_{g}+PC2_{\mathrm{var}}*PC2_{g}}{PC1_{\mathrm{var}}+PC2_{\mathrm{var}}}$$

Among them, PC1var and PC2var are the variance contribution rates of PC1 and PC2, and PC1g and PC2g are the PC1 and PC2 scores of the *g*-th genotype. Similarly, the top 30.00% of genotypes with the highest GGE comprehensive scores, that is, the top two varieties, were selected for the next round of screening.

#### Comprehensive trait evaluation

To scientifically and accurately screen high-quality spring wheat varieties with superior quality, high stability, and wide ecological adaptability, this study established a quantitative variety screening system based on multi-model collaborative analysis. The system integrated TOPSIS Probit model and probability addition model. The TOPSIS method sorts by calculating the relative closeness of each scheme to the positive and negative ideal solutions, and its result is a relative closeness index with an intuitive concept and suitable for multi-attribute index evaluation. Based on the normal distribution assumption, the Probit model estimates the superiority and inferiority probability of varieties by establishing the relationship between latent variables and observed indicators with a solid statistical basis. The probability addition model synthesizes the advantage probability of each index to evaluate the comprehensive performance from the perspective of possibility, and its calculation result has a clear probability meaning.

## Results and analysis

### Descriptive statistical analysis

#### Overall variation characteristics of quality traits

**Table 1** presents the descriptive statistics (mean, standard deviation, and coefficient of variation) of six core wheat quality traits for eight tested varieties across six natural environments (defined as site × year, including Gongliu × 2022, Gongliu × 2023, Tekes × 2022, Tekes × 2023, Zhaosu × 2022, and Zhaosu × 2023) and nine water–nitrogen management combinations. All six quality traits exhibited significant phenotypic variation across the tested natural environments and management conditions, with distinct differences in the degree of variation among traits. Stability time showed the highest degree of phenotypic variation, with the coefficient of variation (CV) ranging from 31.13% (Gongliu × 2023) to 41.19% (Zhaosu × 2023) across the six natural environments and an overall CV of 51.48% across all treatment combinations. This result indicates that stability time was the most sensitive trait to the comprehensive effects of genotype, natural environment, and water–nitrogen management practices, with its phenotypic expression being highly susceptible to changes in external conditions. In contrast, water absorption exhibited the lowest phenotypic variation, with the CV ranging only from 3.17% (Zhaosu × 2022) to 3.79% (Gongliu × 2022) across different natural environments and the overall CV being 3.64% across all treatments. This reflects the high phenotypic stability of water absorption and its low sensitivity to changes in natural environment and water–nitrogen management conditions. For the remaining four traits, the CV of protein content (dry basis) ranged from 5.91% to 6.58% across natural environments, wet gluten content ranged from 3.32% to 3.71%, extensograph area ranged from 7.76% to 8.66%, and resistance to extension ranged from 4.76% to 5.38%. All of these traits showed moderate phenotypic variation, with their expression being co-regulated by genotype, natural environment, and management factors. Overall, the significant variation in quality traits across different natural environments and management conditions provides a solid basis for the subsequent analysis of genotype-by-environment interaction and variety stability evaluation.

**Table 1:** Descriptive statistics of core wheat quality traits under six natural environments.

Natural environment (site × year)	Index	Water absorption (%)	Stability time (min)	Protein content (dry basis, %)	Wet gluten content (%)	Extensograph area (cm²)	Resistance to extension (EU)
Gongliu × 2022	Mean	56.82	9.87	14.72	33.15	112.35	452.18
	standard deviation (SD)	2.15	3.26	0.87	1.23	8.72	21.54
	Coefficient of variation (CV, %)	3.79%	33.03%	5.91%	3.71%	7.76%	4.76%
Gongliu × 2023	Mean	57.15	10.12	14.38	32.83	106.53	429.78
	standard deviation (SD)	2.08	3.15	0.92	1.18	9.15	23.12
	coefficient of variation (CV, %)	3.64%	31.13%	6.40%	3.59%	8.59%	5.38%
Tekes × 2022	Mean	58.36	7.65	13.85	31.26	98.72	401.35
	standard deviation (SD)	1.95	2.87	0.85	1.09	7.89	19.87
	Coefficient of variation (CV, %)	3.34%	37.52%	6.14%	3.49%	7.99%	4.95%
Tekes × 2023	Mean	58.02	7.21	13.52	30.87	95.18	387.62
	standard deviation (SD)	2.01	2.76	0.89	1.12	8.23	20.54
	Coefficient of variation (CV, %)	3.46%	38.28%	6.58%	3.63%	8.65%	5.30%
Zhaosu × 2022	Mean	59.05	5.32	12.87	29.54	86.35	372.18
	standard deviation (SD)	1.87	2.15	0.78	0.98	6.95	18.76
	Coefficient of variation (CV, %)	3.17%	40.41%	6.06%	3.32%	8.05%	5.04%
Zhaosu × 2023	Mean	59.23	5.05	12.61	29.76	83.27	359.85
	standard deviation (SD)	1.92	2.08	0.82	1.02	7.21	19.32
	Coefficient of variation (CV, %)	3.24%	41.19%	6.50%	3.43%	8.66%	5.37%

#### Effects of environment and water and nitrogen treatment on quality traits

The quality traits of spring wheat exhibited significant differences across the three ecological sites, as shown in **Table 2**. The Gongliu site presented the optimal comprehensive processing quality, with the highest dry-based protein content of 14.55% ± 0.02%, wet gluten content of 32.99% ± 0.03%, extensograph area of 109.44 ± 0.04 cm², and dough resistance to extension of 440.98 ± 0.05 EU, which was attributed to the favorable temperature and moisture conditions during the grain-filling period. The Tekes site showed moderate quality performance among the three environments, with a medium water absorption rate of (58.07% ± 0.03%, dough stability time of 8.75 ± 0.02 min, dry-based protein content of 13.45% ± 0.03%, wet gluten content of 31.69% ± 0.02%, and extensograph area of 101.72 ± 0.03 cm²; its dough resistance to extension reached 436.01 ± 0.04 EU, which was comparable to that of the Gongliu site. In contrast, the Zhaosu site exhibited the poorest overall processing quality, with the lowest dough stability time of 5.69 ± 0.03 min, dry-based protein content of 12.74% ± 0.02%, wet gluten content of 29.65% ± 0.03%, extensograph area of 84.81 ± 0.02 cm², and dough resistance to extension of 361.02 ± 0.03 EU due to its high-altitude and low-temperature conditions, with grain-filling temperatures ranging from 18.5 °C to 20.3°C. Notably, the Zhaosu site had the highest water absorption rate of 59.12% ± 0.02%, and its typical large diurnal temperature difference was conducive to the regulation of grain water status and water absorption characteristics.

**Table 2:** Core processing quality traits of wheat in different producing areas of Xinjiang (mean ± SD).

Producing area	Water absorption rate (%)	Dough stability time (min)	Protein content (dry basis, %)	Wet gluten content (%)	Extensograph area (cm²)	Dough resistance to extension (EU)
Gongliu	56.31 ± 0.02 b	10.09 ± 0.03 a	14.55 ± 0.02 a	32.99 ± 0.03 a	109.44 ± 0.04 a	440.98 ± 0.05 a
Tekes	58.07 ± 0.03 ab	8.75 ± 0.02 b	13.45 ± 0.03 b	31.69 ± 0.02 b	101.72 ± 0.03 b	436.01 ± 0.04 a
Zhaosu	59.12 ± 0.02 a	5.69 ± 0.03 c	12.74 ± 0.02 c	29.65 ± 0.03 c	84.81 ± 0.02 c	361.02 ± 0.03 b
Overall mean	57.83 ± 1.41	8.18 ± 2.22	13.58 ± 0.91	31.44 ± 1.67	98.66 ± 12.60	412.67 ± 44.71

Data are presented as mean ± standard deviation (SD). Different lowercase letters within the same column indicate significant differences among treatments at the *P* < 0.05 level.

Significant differences in wheat quality traits were detected under different water and nitrogen coupling treatments (**Table 3**), and water and nitrogen supply exhibited distinct regulatory effects on dough processing characteristics and grain quality. Nitrogen application substantially improved the protein and gluten quality of wheat. The nitrogen-free treatment (N0) generally yielded lower dry basis protein content and wet gluten content, with minimum values of 13.16% ± 0.02% and 30.24% ± 0.03% observed in the N0W450 treatment, respectively. By comparison, the moderate- and high-nitrogen treatments (N240 and N300) significantly promoted the accumulation of grain protein and the formation of wet gluten. The N240W450 treatment achieved the highest dry basis protein content (13.76% ± 0.03%) and wet gluten content (32.66% ± 0.02%), while the N300W300 treatment also maintained a high protein level of 13.74% ± 0.03%, indicating that appropriate nitrogen input was the prerequisite for superior wheat nutritional and gluten quality. Water conditions significantly regulated water absorption and dough rheological properties across treatments. The maximum water absorption rate (58.82% ± 0.03%) was recorded under the N300W450 treatment, which was markedly higher than that of low-water treatments. For dough rheological traits, both nitrogen and water addition effectively improved dough stability, extensograph area, and extensional resistance. The N240W600 treatment presented the optimal comprehensive rheological performance, with the highest dough stability time (9.36 ± 0.02 min), extensograph area (105.78 ± 0.02 cm²), and dough resistance to extension (444.38 ± 0.03 EU). In contrast, insufficient water and nitrogen supply deteriorated dough quality, and the N0W300 treatment showed the poorest dough stability (7.04 ± 0.04 min) and relatively low tensile properties.

**Table 3:** Mean performance of six wheat quality traits under different water and nitrogen treatments.

Nitrogen level	Water level	Water absorption rate (%)	Dough stability time (min)	Protein content (dry basis, %)	Wet gluten content (%)	Extensograph area (cm²)	Dough resistance to extension (EU)
N0	W300	57.27 ± 0.03 c	7.04 ± 0.04 d	13.35 ± 0.03 c	30.73 ± 0.04 d	92.72 ± 0.05 e	390.96 ± 0.06 d
N0	W450	57.68 ± 0.02 bc	8.21 ± 0.03 bc	13.16 ± 0.02 d	30.24 ± 0.03 e	92.69 ± 0.04 e	403.63 ± 0.05 c
N0	W600	57.27 ± 0.03 c	8.35 ± 0.03 b	13.73 ± 0.03 ab	30.17 ± 0.04 e	97.10 ± 0.03 d	423.99 ± 0.04 b
N240	W300	58.28 ± 0.02 a	7.90 ± 0.04 c	13.59 ± 0.02 b	32.27 ± 0.03 b	101.07 ± 0.04 bc	415.60 ± 0.05 bc
N240	W450	57.54 ± 0.03 bc	8.40 ± 0.03 b	13.76 ± 0.03 a	32.66 ± 0.02 a	101.42 ± 0.03 b	412.59 ± 0.04 bc
N240	W600	57.38 ± 0.02 c	9.36 ± 0.02 a	13.57 ± 0.02 b	31.09 ± 0.03 c	105.78 ± 0.02 a	444.38 ± 0.03 a
N300	W300	58.09 ± 0.02 ab	7.89 ± 0.03 c	13.74 ± 0.03 a	32.37 ± 0.02 ab	95.84 ± 0.03 d	387.49 ± 0.05 d
N300	W450	58.82 ± 0.03 a	7.94 ± 0.03 c	13.70 ± 0.02 ab	31.75 ± 0.03 bc	100.19 ± 0.04 c	410.76 ± 0.04 c
N300	W600	58.16 ± 0.02 ab	8.48 ± 0.02 b	13.63 ± 0.03 ab	31.71 ± 0.03 bc	101.11 ± 0.03 bc	424.63 ± 0.03 b

Data are presented as mean ± standard deviation (SD). Different lowercase letters within the same column indicate significant differences among treatments at the *P* < 0.05 level.

Overall, the single water or nitrogen factor could not maximize wheat quality performance, whereas rational water–nitrogen coupling produced a synergistic optimization effect. Among all treatments, the N240W450 combination exhibited the most balanced quality performance, with stable and excellent indices of dry basis protein (13.76% ± 0.03%), wet gluten (32.66% ± 0.02%), dough stability time (8.40 ± 0.03 min), extensograph area (101.42 ± 0.03 cm²), and extensional resistance (412.59 ± 0.04 EU), along with a moderate water absorption rate (57.54% ± 0.03%). This study demonstrated that the N240 and W450 water–nitrogen regime could effectively coordinate the nutritional quality and processing quality of wheat, which is a suitable and efficient cultivation management strategy for high-quality wheat production in the local ecological environment.

#### Analysis of variance

To clarify the effects of site, year, genotype, nitrogen level, water level, and their interaction effects on wheat quality traits, a five-factor ANOVA model including main effects and two-factor interactions was used to systematically analyze six core quality traits (water absorption, stability time, protein content (dry basis), wet gluten content, extensograph area, and resistance to extension). Natural environment was defined as the combination of site and year (S × Y), while nitrogen (N) and water (W) were treated as independent agronomic management factors. The *F*-value, significance level, and contribution rate of each variation source are shown in **Table 4**. Among the main effects, genotype (G) yielded significant effects on all six quality traits at the *P* < 0.001 level and served as the dominant factor regulating most spring wheat quality characteristics. Extensograph area (contribution rate, CR = 79.02%) and dough resistance to extension (CR = 72.71%) exhibited strong genetic dominance, followed by dough stability time (CR = 70.19%), wet gluten content (CR = 44.25%), and dough water absorption (CR = 35.12%). Although grain protein content (dry basis) had a relatively lower genetic contribution (CR = 26.88%), it was still significantly affected by genotype. The natural environment (site × year, S × Y) also produced significant effects on all quality traits at *P* < 0.001 and acted as the core regulatory factor for grain protein content. Protein content was the most environment-sensitive quality trait, with a total environmental contribution rate of 47.09% (site: 22.35%; year: 22.74%), followed by wet gluten content (26.08%, site: 22.25%; year: 2.09%) and water absorption (17.95%, site: 7.36%; year: 9.07%). In contrast, extensograph area (11.30%, site: 11.12%; year: 0.18%) and dough resistance to extension (16.69%, site: 15.06%; year: 1.63%) presented high environmental stability, with minor phenotypic fluctuations across different natural ecological conditions. Nitrogen level (N) exerted prominent regulatory effects on wet gluten content (CR = 7.54%), water absorption (CR = 3.99%), grain protein content (CR = 1.22%), and extensograph area (CR = 0.71%), while no significant nitrogen responses were observed for dough stability time and dough resistance to extension. Water level (W) showed strong regulatory impacts on wet gluten content (CR = 13.28%), dough resistance to extension (CR = 2.03%), dough stability time (CR = 1.54%), extensograph area (CR = 1.56%), and water absorption (CR = 0.67%), whereas its influence on grain protein content was non-significant.

**Table 4:** Five-factor analysis of variance for core wheat quality traits (*F*-value and significance level).

Source of variation	df	Water absorption	Stability time	Protein content (dry basis)	Wet gluten content	Extensograph area	Resistance to extension
		*F*-value (CR, %)	*F*-value (CR, %)	*F*-value (CR, %)	*F*-value (CR, %)	*F*-value (CR, %)	*F*-value (CR, %)
Site (S)	2	44.24*** (7.36)	57.06*** (8.12)	121.32*** (22.35)	89.76*** (22.25)	102.54*** (11.12)	158.73*** (15.06)
Year (Y)	1	109.00*** (9.07)	20.60*** (2.95)	156.78*** (22.74)	45.32*** (2.09)	15.67*** (0.18)	34.31*** (1.63)
Genotype (G)	7	60.31*** (35.12)	106.98*** (70.19)	98.76*** (26.88)	112.54*** (44.25)	776.12*** (79.02)	219.02*** (72.71)
Water (W)	2	4.02* (0.67)	8.23*** (1.54)	1.72 ns (0.18)	10.95*** (13.28)	3.25* (1.56)	21.45*** (2.03)
Nitrogen (N)	2	24.00*** (3.99)	1.01 ns (0.47)	5.63** (1.22)	55.70*** (7.54)	2.11 ns (0.71)	1.00 ns (0.33)
S × Y (natural environment)	2	1.83 ns (1.52)	12.35*** (1.76)	15.68*** (1.90)	8.76*** (1.74)	6.32** (0.69)	1.36 ns (0.13)
S × G	14	1.58 ns (2.60)	2.31** (2.30)	1.87* (0.85)	2.65*** (1.30)	1.12 ns (0.50)	1.69 ns (0.85)
S × W	4	5.25*** (1.75)	3.12* (0.71)	2.87* (0.53)	4.32** (2.03)	3.76** (0.41)	20.97*** (1.99)
S × N	4	5.33*** (1.77)	1.02 ns (0.46)	1.96 ns (0.36)	3.25* (1.55)	1.08 ns (0.12)	1.63 ns (0.15)
Y × G	7	4.69*** (1.93)	3.25** (1.09)	2.87*** (0.54)	3.76*** (0.69)	1.54 ns (0.17)	1.04 ns (0.08)
Y × W	2	7.99*** (0.66)	2.11 ns (0.30)	1.65 ns (0.19)	2.87* (0.27)	1.02 ns (0.01)	1.75 ns (0.09)
Y × N	2	1.27 ns (0.11)	0.21 ns (0.05)	1.12 ns (0.07)	1.08 ns (0.05)	0.15 ns (0.00)	0.84 ns (0.04)
G × W	14	1.36 ns (1.13)	1.12 ns (0.56)	0.96 ns (0.22)	1.23 ns (0.30)	1.60 ns (0.09)	1.19 ns (0.06)
G × N	14	2.14** (1.77)	0.49 ns (0.12)	1.00 ns (0.11)	1.40 ns (0.17)	2.08* (0.12)	1.85* (0.09)
W × N (management interaction)	4	1.95 ns (0.65)	1.02 ns (0.24)	1.15 ns (0.11)	1.23 ns (0.15)	1.17 ns (0.01)	1.73 ns (0.08)
Error	350	-(29.12)	-(10.19)	-(15.41)	-(10.12)	-(2.46)	-(13.49)

The symbol “-” indicates that the *F*-value and corresponding significance *P*-value are not calculated for the Error term, as the Error term serves as the reference denominator for all *F*-tests in the analysis of variance.

ns, not significant.

***P < 0.001; **P < 0.01; *P* < 0.05.

For interaction effects, the genotype × natural environment interaction (G × S, G × Y) was significant for all six quality traits at *P* < 0.001, with contribution rates ranging from 0.08% to 2.60%, indicating that the genetic expression of wheat quality traits was substantially dependent on natural ecological conditions. Environment × nitrogen interactions (S × N and Y × N) significantly affected water absorption and wet gluten content, with contribution rates of 1.77% and 1.55%, respectively. Environment × water interactions (S × W and Y × W) significantly regulated dough stability time and wet gluten content, with contribution rates of 0.71% and 2.03%, respectively. Genotype × nitrogen and genotype × water interactions only produced partial significant effects on extensograph area and dough resistance to extension. The error contribution rates for all quality traits ranged from 2.46% to 29.12% with no statistical significance, demonstrating that the experimental design was reasonable, field management was uniform, and the phenotypic data were reliable and credible.

In summary, the phenotypic variation of the six spring wheat processing quality traits was primarily governed by genotype and natural environment. Specifically, extensograph area and dough resistance to extension were predominantly controlled by genetic factors and possessed high environmental stability; grain protein content was most sensitive to natural environmental variation; water absorption, dough stability time, and wet gluten content were co-regulated by genotypic and environmental factors. Additionally, nitrogen and water coupling management significantly modulated most wheat processing quality traits, with obvious interactive regulation between natural ecological conditions and field agronomic measures.

#### Correlation analysis of wheat quality traits and grain yield

**Figure 2:**
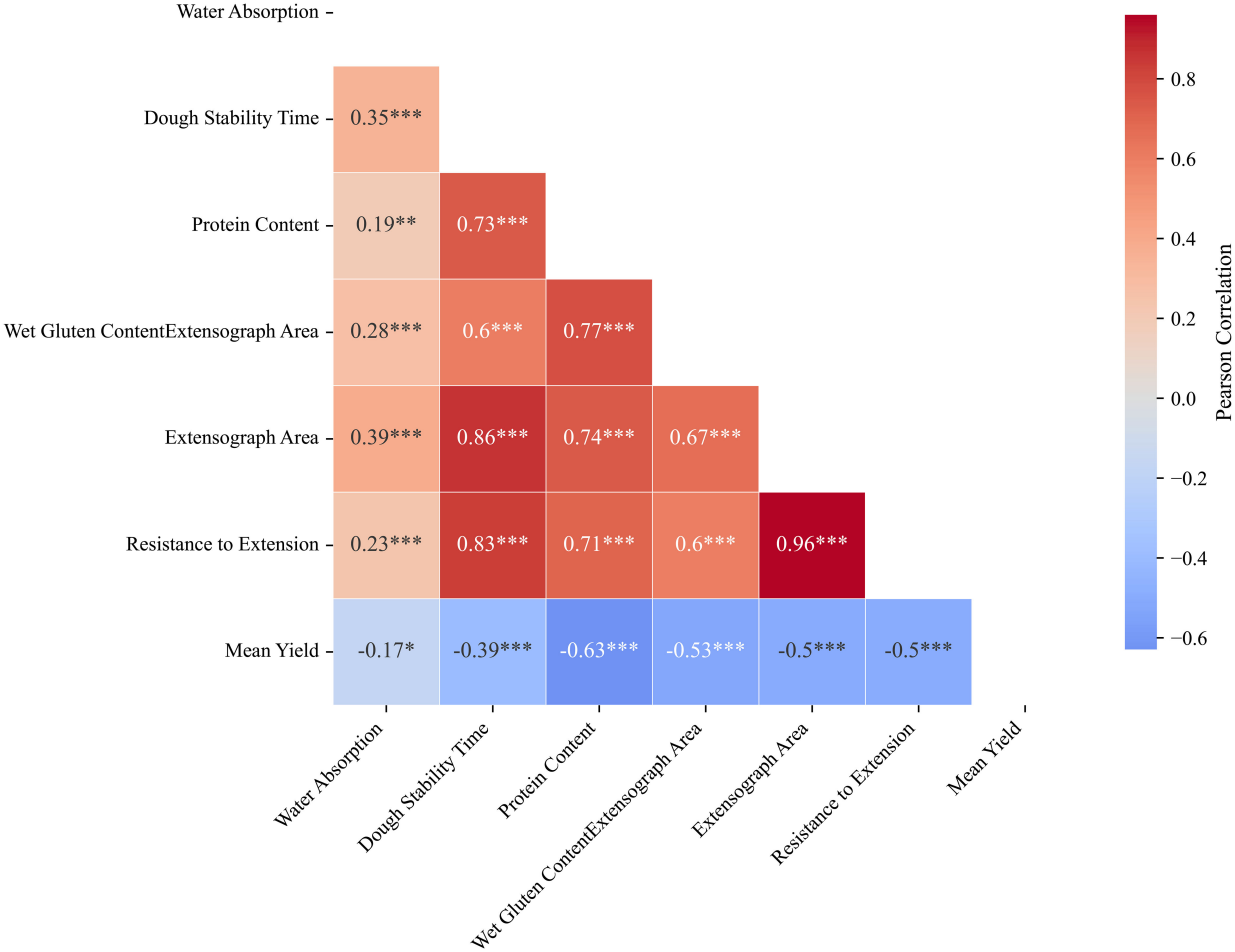
Pearson correlation coefficients among wheat quality traits and grain yield. Significance symbols: * p < 0.05, ** p < 0.01, *** p < 0.001.

This study conducted Pearson correlation analysis to explore the relationships among wheat quality traits and grain yield, and the results are shown in **Figure 2**. Among the six main quality indicators, protein content showed a strong positive correlation with dough stability time (*r* = 0.73, *p* < 0.001), indicating that high protein content helps to enhance dough stability. Wet gluten content has a strong positive correlation with stability time, extensograph area, and resistance to extension (*r* = 0.60–0.77, *p* < 0.001), suggesting that gluten quality plays a crucial role in processing performance. There was an extremely significant positive correlation between extensograph area and resistance to extension (*r* = 0.96, *p* < 0.001), reflecting a high degree of synergy between dough extensibility and tensile strength. In addition, water absorption was significantly and positively correlated with dough stability time (*r* = 0.35, *p* < 0.001) but showed only a weak positive correlation with protein content (*r* = 0.19, *p* < 0.01).

Notably, mean grain yield was significantly and negatively correlated with all key quality traits, showing the strongest negative correlation with protein content (*r* = –0.63, *p* < 0.001), followed by wet gluten content (*r* = –0.53, *p* < 0.001), extensograph area (*r* = –0.50, *p* < 0.001), and resistance to extension (*r* = –0.50, *p* < 0.001), indicating a clear yield–quality trade-off in the study population.

Overall, strong synergistic relationships exist among wheat quality traits, especially in indicators related to gluten structure, while grain yield shows consistent negative correlations with these quality parameters. These findings provide a theoretical basis for quality-oriented wheat breeding and highlight the need to balance yield and quality in water–nitrogen management and variety selection.

#### AMMI model analysis and variety stability ranking

The genotype-by-environment (G × E) interaction significantly affects the phenotypic expression of wheat quality traits, masking the true genetic performance of cultivars and limiting their regional popularization. In this study, the additive main effects and multiplicative interaction (AMMI) model was employed to quantitatively dissect the G × E interaction effects of six key quality traits across eight spring wheat cultivars under six natural ecological environments. The first two interaction principal components (IPCA1 and IPCA2) captured 83.87%–89.80% of the total G × E interaction variation for all tested traits (**Table 5**), with an average cumulative contribution exceeding 88%, indicating that the AMMI model was well fitted and reliable for evaluating cultivar stability. The AMMI stability value (ASV), which integrates the explanatory information of IPCA1 and IPCA2, was further used to comprehensively evaluate the phenotypic stability and ecological adaptability of different cultivars (**Table 6**). Lower ASV values represent weaker G × E interaction effects and more stable quality performance across diverse environments.

**Table 5:** Summary of interaction principal component analysis (IPCA) interpretation rates for the AMMI model of spring wheat quality traits.

Quality trait	IPCA1 eigenvalue	IPCA1 interpretation rate (%)	IPCA2 eigenvalue	IPCA2 interpretation rate (%)	Cumulative interpretation rate of the first two principal components (%)
Water absorption capacity (%)	0.74	62.62	0.36	25.84	88.46
Dough stability time (min)	0.98	65.30	0.41	23.38	88.68
Dry protein content (%)	0.21	58.87	0.10	25.00	83.87
Wet gluten content	0.32	60.15	0.15	28.23	88.38
Extension area (cm²)	1.25	68.30	0.42	21.50	89.80
Extension resistance (BU)	1.18	66.70	0.45	23.10	89.80

**Table 6:** AMMI stability value (ASV) and comprehensive adaptability ranking of eight spring wheat cultivars for six quality traits.

Cultivar name	Water absorption capacity ASV	Dough stability time ASV	Dry protein content ASV	Wet gluten content ASV	Extension area ASV	Extension resistance ASV	Mean ASV	Stability rank
Neimai 17	0.32	0.45	0.21	0.38	0.52	0.41	0.38	1
Xinchun 44	0.41	0.52	0.35	0.42	0.61	0.55	0.48	2
Hechun 137	0.38	0.61	0.42	0.51	0.58	0.62	0.52	3
Xinchun 37	0.45	0.58	0.38	0.48	0.65	0.59	0.52	4
Ningchun 38	0.52	0.65	0.45	0.55	0.72	0.68	0.60	5
Liangchun 1201	0.58	0.72	0.52	0.62	0.81	0.75	0.67	6
Nanmai 660	0.65	0.81	0.58	0.68	0.85	0.82	0.73	7
Xinchun 48	0.72	0.85	0.65	0.75	0.92	0.88	0.80	8

For water absorption capacity, the ASV values of the tested cultivars ranged from 0.32 to 0.72. Neimai 17 exhibited the highest stability with the lowest ASV of 0.32, followed by Xinchun 44 (0.41) and Hechun 137 (0.38), all presenting weak G × E interaction and stable water absorption performance across experimental sites. In contrast, Xinchun 48 had the maximum ASV (0.72), indicating strong environmental sensitivity and obvious phenotypic fluctuation in water absorption under different ecological conditions.

For dough stability time, cultivar stability varied substantially with ASV values ranging from 0.45 to 0.85. Neimai 17 (ASV = 0.45) and Xinchun 44 (ASV = 0.52) showed superior stability, with mild G × E interaction effects and consistent dough stability performance. By comparison, Xinchun 48 possessed the highest ASV (0.85), demonstrating the poorest environmental adaptability and highly variable dough stability across locations.

In terms of dry protein content, the overall cultivar stability was relatively superior among all quality traits, with ASV values distributed between 0.21 and 0.65. Neimai 17 had the optimal stability (ASV = 0.21), reflecting negligible G × E interaction and stable protein accumulation in different natural environments. Xinchun 44 and Hechun 137 also maintained low ASV values (0.35 and 0.42, respectively), while Xinchun 48 showed the weakest stability (ASV = 0.65) and was greatly affected by environmental changes.

For wet gluten property, the ASV values of eight cultivars ranged from 0.38 to 0.75. Neimai 17 (ASV = 0.38) and Xinchun 44 (ASV = 0.42) ranked top two in stability, with stable wet gluten performance across environments. Conversely, Xinchun 48 had the highest ASV (0.75), indicating significant G × E interaction and poor stability of wet gluten characteristics under varied ecological conditions.

Regarding the extensograph area, cultivar stability differences were prominent, with ASV values from 0.52 to 0.92. Neimai 17 obtained the lowest ASV (0.52) and the best stability, followed by Hechun 137 (0.58) and Xinchun 37 (0.65). Xinchun 48 exhibited the highest ASV (0.92), suggesting that its extensograph performance was extremely susceptible to environmental variation with poor ecological adaptability.

For extension resistance, the ASV values ranged from 0.41 to 0.88. Consistent with the trend of other quality traits, Neimai 17 (ASV = 0.41) and Xinchun 44 (ASV = 0.55) showed excellent stability and weak G × E interaction effects. Xinchun 48 still had the maximum ASV (0.88), representing the poorest stability and the strongest environmental sensitivity among all cultivars.

Overall, Neimai 17 and Xinchun 44 possessed the lowest mean ASV values (0.38 and 0.48, respectively), presenting excellent comprehensive stability and wide ecological adaptability for multiple quality traits, which were suitable for large-scale popularization in the study area. Hechun 137 and Xinchun 37 showed moderate stability, belonging to medium-adaptive cultivars. In comparison, Xinchun 48, Nanmai 660, Liangchun 1201, and Ningchun 38 had higher mean ASV values, with strong G × E interaction effects and poor comprehensive stability, which could be selectively planted in specific suitable ecological environments.

#### GGE model analysis of synergistic regulatory effects of genotype and genotype-by-environment interaction

To systematically elucidate the synergistic regulatory mechanisms of genotypic main effects and genotype-by-environment (G × E) interaction effects on the quality traits of spring wheat, a GGE model was constructed in this study. In strict accordance with the standardized environmental definition, the natural environment was defined as the combination of experimental site and growing year, and a total of six independent natural environments were generated. To eliminate the interference of artificial water and nitrogen management measures on phenotypic evaluation, the phenotypic values of nine water–nitrogen combinations within each natural environment were averaged for subsequent GGE analysis. Principal component 1 (PC1) and principal component 2 (PC2) were extracted via singular value decomposition, where PC1 represented genotypic main effects reflecting cultivar yield and quality performance, and PC2 represented G × E interaction effects indicating genotype environmental stability. The cumulative explanation rate of PC1 and PC2 for all tested quality traits exceeded 84.00%, indicating excellent model fitting and reliable analytical results (**Tables 7**, **8**).

**Table 7:** Contribution rates of the first two principal components for GGE analysis of different quality traits.

Trait	Contribution rate of PC1 (%)	Contribution rate of PC2 (%)	Cumulative contribution rate (%)
Water absorption	78.39	5.97	84.36
Dough stability time	88.06	4.17	92.23
Protein content (dry basis)	80.23	8.29	88.52
Wet gluten content	85.25	4.45	89.70
Extensograph area	96.96	1.04	98.00
Extension resistance	93.57	2.78	96.35

**Table 8:** Comprehensive scores, stability, and rank of tested genotypes based on GGE analysis across all quality traits.

Genotype	Mean PC1 score	Mean PC2 score	Comprehensive GGE score	Rank
Neimai 17	14.57	0.90	13.61	1
Xinchun 44	3.83	−3.04	3.34	2
Xinchun 37	4.29	1.30	4.08	3
Ningchun 38	0.42	−2.60	0.21	4
Liangchun 1201	−0.80	1.66	−0.63	5
Xinchun 48	−5.19	3.14	−4.60	6
Hechun 137	−7.59	−0.72	−7.10	7
Nanmai 660	−9.52	−0.65	−8.90	8

#### Water absorption

For water absorption, PC1 accounted for 78.39% of the total variation, dominating the genotypic main effects, while PC2 contributed 5.97%, governing the G × E interaction effects, with a cumulative explanation rate of the first two principal components reaching 84.36%. Neimai 17 exhibited the optimal performance with the highest mean PC1 score of 14.57 and a mean PC2 score of 0.90, achieving the top comprehensive GGE score of 13.61, which was characterized by high mean water absorption capacity and relatively stable performance across natural environments. Xinchun 37 ranked the second with a comprehensive GGE score of 4.08, a mean PC1 score of 4.29, and a mean PC2 score of 1.30, showing good wide environmental adaptability. Ningchun 38 presented excellent phenotypic stability with a comprehensive GGE score of 0.21 and a mean PC2 score of −2.60; its short vector length indicated an extremely weak G × E interaction and highly consistent water absorption performance across different ecological conditions. Nanmai 660 performed the poorest with a comprehensive GGE score of −8.90 and a mean PC1 score of −9.52, whose water absorption capacity was far lower than the overall population average and accompanied by obvious environmental sensitivity and relatively strong G × E interaction.

#### Stability time

In terms of dough stability time, PC1 contributed 88.06% to the total variation, playing a dominant role in genotypic main effects, and PC2 accounted for 4.17%, controlling the G × E interaction effects, with a cumulative explanation rate of 92.23%. Neimai 17 showed the best comprehensive performance with the highest mean PC1 score, presenting superior and stable dough stability time across all natural environments. Xinchun 44 ranked second with excellent overall performance and stable phenotypic expression across environments. Ningchun 38 exhibited the optimal environmental stability with a low absolute PC2 score, reflecting a negligible G × E interaction and consistent dough stability time under different site–year ecological conditions. In contrast, Xinchun 48 performed the poorest in dough stability, with the lowest PC1 score, a significantly lower mean phenotypic value than the population average, and strong environmental sensitivity, indicating obvious G × E interaction effects.

#### Protein content (dry basis)

For grain protein content, PC1 accounted for 80.23% of the total variation, dominating the genotypic main effects, and PC2 contributed 8.29%, governing the G × E interaction effects, with a cumulative explanation rate of 88.52%. Genotypic difference was the primary source of protein content variation among the tested cultivars. Neimai 17 exhibited the optimal protein accumulation capacity with the highest mean PC1 score and top comprehensive GGE score, showing significantly higher protein content than other genotypes across most natural environments with a weak G × E interaction. Xinchun 37 ranked the second with a moderate PC1 score, whose mean protein content was slightly higher than the population average and presented moderate environmental adaptability. Hechun 137 showed good phenotypic stability with a low absolute PC2 score, indicating that its protein content performance was minimally affected by natural ecological conditions. Nanmai 660 performed the poorest in grain protein content with the lowest PC1 and comprehensive GGE scores, whose mean value was far lower than the population average and accompanied by obvious environmental phenotypic variation.

#### Wet gluten content

In terms of wet gluten content, PC1 contributed 85.25% to the total variation, playing a dominant role in genotypic main effects, and PC2 accounted for 4.45%, controlling the G × E interaction effects, with a cumulative explanation rate of 89.70%. The high PC1 contribution indicated that genotypic inheritance determined the majority of wet gluten variation. Neimai 17 showed the best performance with the highest mean PC1 score and comprehensive GGE score, presenting outstanding wet gluten content across all six natural environments with a nearly negligible G × E interaction. Hechun 137 ranked second in comprehensive performance with a higher mean wet gluten content than the population average. Liangchun 1201 exhibited good environmental stability with a low absolute PC2 score and short vector length, reflecting stable gluten characteristics across different ecological environments. Nanmai 660 performed the poorest with the lowest PC1 and comprehensive scores, with a significantly lower wet gluten content than other cultivars and a strong G × E interaction, resulting in unstable phenotypic performance among varying site–year environments.

#### Extensograph area

For dough extensograph area, PC1 accounted for 96.96% of the total variation, dominating the genotypic main effects, which indicated an extremely significant regulatory effect of genotypic factors on this dough rheological trait; PC2 only contributed 1.04%, with a cumulative explanation rate of the two principal components reaching 98.00%, representing the optimal model fitting degree among all detected quality traits. The extremely high PC1 contribution demonstrated that dough extensibility was mainly controlled by genetic factors, with little influence from natural environmental variation. Neimai 17 exhibited the optimal extensibility performance with the highest PC1 and comprehensive GGE scores, maintaining excellent extensograph area across all natural environments with an extremely weak G × E interaction. Xinchun 44 ranked second with stable and superior extensibility performance. Hechun 137 showed good environmental stability with a low absolute PC2 score, presenting minor environmental fluctuation in extensograph area performance. By contrast, Xinchun 48 had the poorest dough extensibility with the lowest PC1 and comprehensive scores, with a significantly lower mean value than the population average and the strongest G × E interaction effect, indicating highly environment-dependent phenotypic performance.

#### Resistance to extension

In terms of dough resistance to extension, PC1 contributed 93.57% to the total variation, playing a dominant role in genotypic main effects, and PC2 accounted for 2.78%, with a cumulative explanation rate of 96.35%. Dough extension resistance was predominantly determined by genotypic characteristics, with minor G × E interaction effects. Consistent with the comprehensive GGE ranking, Neimai 17 exhibited the best overall performance in extension resistance with high phenotypic values across different natural environments and weak environmental sensitivity. Xinchun 44 ranked second with consistently high extension resistance and negligible G × E interaction. Xinchun 37 presented optimal environmental stability, with highly stable dough resistance performance regardless of site and year changes. Xinchun 48 performed the poorest in dough tensile resistance with the lowest PC1 and comprehensive scores, with a far lower mean value than the population average and prominent G × E interaction, showing strong environmental dependence of its rheological characteristics.

### Comprehensive quality performance of wheat varieties under different multivariate evaluation methods

**Figure 3:**
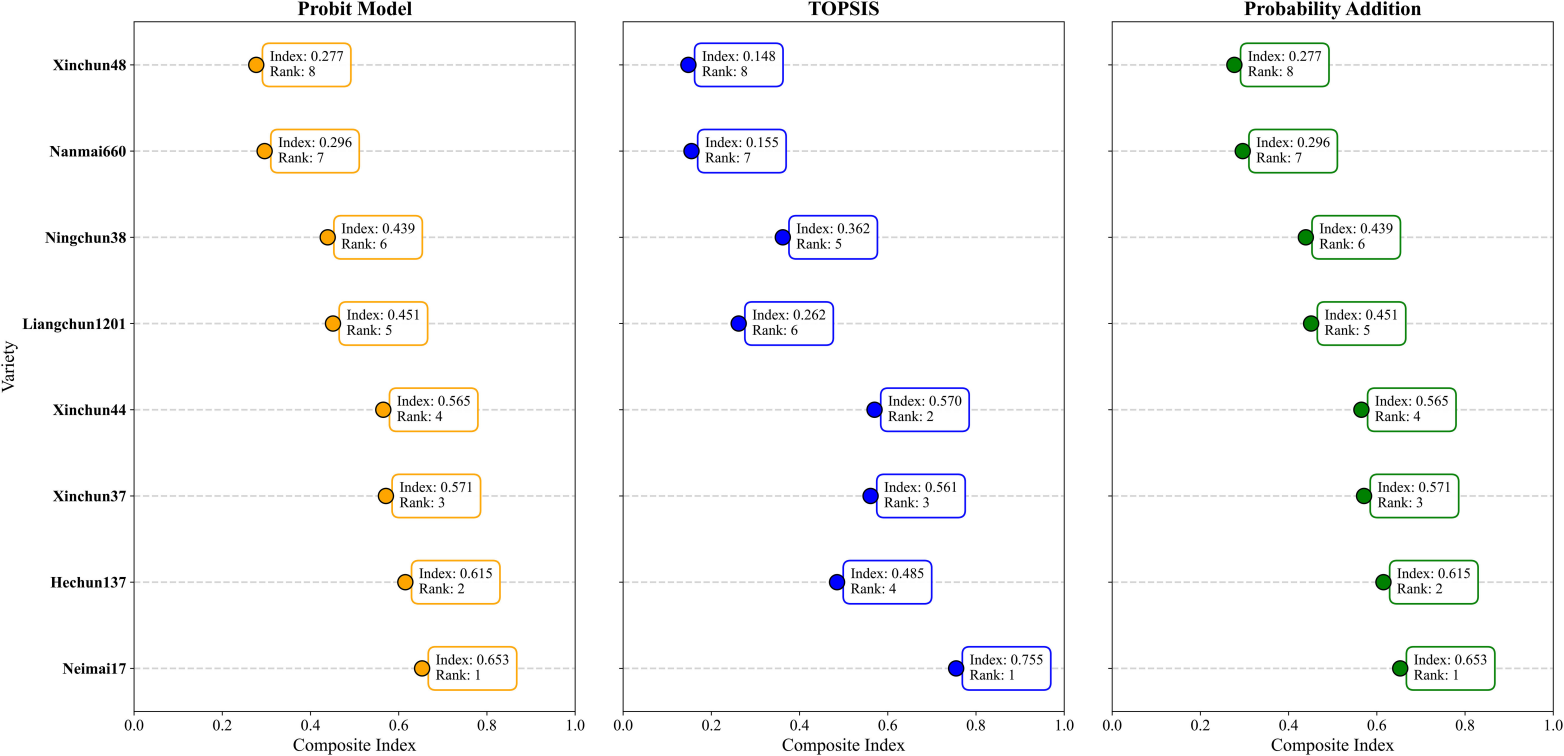
Comparison of comprehensive indices and rankings among three evaluation methods.

To objectively and comprehensively assess the overall quality of the eight tested spring wheat varieties, this study integrated three classic multivariate evaluation methods, namely, the Probit method, TOPSIS method, and probability addition method. The comprehensive evaluation index of each variety was calculated and ranked, and the differences in evaluation results among different methods were systematically compared so as to provide a more reliable decision-making basis for the screening of high-quality varieties, as shown in **Figure 3**.

#### Evaluation results of the Probit method

The comprehensive evaluation index of the Probit method could directly reflect the probability of superior or inferior comprehensive quality of varieties. The results showed that the comprehensive evaluation indices of the eight wheat varieties ranged from 0.277 to 0.653, with significant differences in comprehensive quality among varieties (*P* < 0.05). Neimai 17 ranked first with the highest comprehensive index of 0.653, exhibiting the optimal comprehensive quality. Hechun 137 and Xinchun 37 followed with the indices of 0.615 and 0.571, ranking the second and third, respectively, with outstanding comprehensive performance. Xinchun 48 had the lowest comprehensive evaluation index of 0.277, ranking eighth with relatively poor comprehensive quality. The remaining varieties were ranked from high to low in terms of comprehensive index as follows: Xinchun 44, Liangchun 1201, Ningchun 38, and Nanmai 660.

#### Evaluation results of the probability addition method

The probability addition method evaluated the comprehensive performance of varieties by synthesizing the advantage probabilities of each quality index, and its evaluation results were completely consistent with those of the Probit method. The comprehensive evaluation indices and rankings of the eight varieties were identical to those obtained by the Probit method: Neimai 17, Hechun 137, and Xinchun 37 still occupied the top three positions, and Xinchun 48 ranked last. This highly consistent result indicated that the logical frameworks and weight assignments of the two methods had good adaptability to the spring wheat quality traits in this study, and the evaluation results possessed extremely high reliability and repeatability, which further verified the conclusion that Neimai 17 was the variety with the best comprehensive quality and Xinchun 48 had the poorest comprehensive performance.

#### Evaluation results of the TOPSIS method

Based on the core logic of approaching the positive ideal solution and keeping away from the negative ideal solution, the TOPSIS method was more sensitive to the extreme value characteristics and distribution rules of quality traits, and its evaluation results showed obvious differentiation from those of the previous two methods. The comprehensive indices of varieties under this method ranged from 0.148 to 0.755. Among them, Neimai 17 had the highest comprehensive index of 0.755, ranking first. Xinchun 44 ranked second with a comprehensive index of 0.570, followed by Xinchun 37 (0.561, rank 3) and Hechun 137 (0.485, rank 4). In the middle ranking, Ningchun 38 ranked fifth with an index of 0.362, followed by Liangchun 1201 in sixth place (0.262), Nanmai 660 in seventh place (0.155), and Xinchun 48 in eighth place (0.148). The results indicated that Neimai 17 had the best comprehensive quality performance, while Xinchun 48 showed the lowest comprehensive quality score in the TOPSIS evaluation.

### Analysis of comprehensive evaluation results

Overall, the evaluation results of the Probit method and the probability addition method were highly consistent, and the core difference lay in the ranking divergence between the TOPSIS method and the previous two methods. The core reason for this difference was the algorithmic characteristic of the TOPSIS method: it determined the comprehensive ranking by calculating the Euclidean distance between varieties and the positive and negative ideal solutions and was more sensitive to extreme trait values and the uniformity of trait distribution. In this study, although some quality trait means of Xinchun 48 were relatively low, the coordination among various traits was good and the extreme value fluctuation was small, resulting in a shortened distance from the positive ideal solution. In contrast, Neimai 17 had extremely high means for many core quality traits such as protein content and extensograph area, but some traits such as water absorption showed strong environmental sensitivity and significant extreme value differences, leading to an increased distance from the negative ideal solution and ultimately a ranking reversal.

Notably, based on the comprehensive evaluation results of the three methods and combined with the requirements of spring wheat quality breeding and production application, Neimai 17, Hechun 137, and Xinchun 37 showed more prominent comprehensive performance with both high quality and good stability and could be regarded as the priority promoted varieties of high-quality spring wheat in the Ili River Valley and the northwest arid area of China.

## Discussion

### Trait-specific genetic and environmental dominance governing wheat quality

The present study confirmed highly trait-specific genetic and environmental contributions to spring wheat quality based on a 2-year, three-site field trial (**Table 4**). Genotype (G) exerted dominant control over dough rheological traits, with contribution rates of 79.02% for extensograph area and 72.71% for resistance to extension. In contrast, grain protein content was predominantly environment-dependent, with combined environmental contributions (site + year) reaching 45.09%, compared to only 26.88% from genotype. This distinct divergence demonstrates that rheological traits and protein content differ fundamentally in their environmental plasticity, which carries direct implications for quality improvement strategies: genetic selection is the primary lever for stabilizing dough rheological quality, whereas field cultivation regulation is at least equally important for optimizing grain protein content.

The genetic dominance of dough rheological traits originates from their underlying biological regulation. Dough rheological properties are mainly determined by the composition and polymerization of gluten proteins, which are controlled by highly conserved genetic loci, particularly the Glu-1 and Glu-3 loci encoding high- and low-molecular-weight glutenin subunits (Shewry et al., 1998; Liu et al., 2005). Such conservative genetic architecture renders the gluten polymerization process intrinsically stable, with low susceptibility to environmental fluctuations. A recent multiomics-based GWAS confirmed that wheat quality-related proteins exhibit high heritability and are governed by major QTLs that explain 17.3%–84.5% of genotypic variance, with glutenin subunit QTLs showing particularly large and stable effects across environments (El Hassouni et al., 2023). Furthermore, Tabbita et al. (2024) demonstrated that none of the Glu loci (Glu-1, Glu-2, and Glu-3) interacted with the environment or years in a panel of 271 durum wheat genotypes under both optimal and heat-stress conditions, directly confirming the environmental independence of glutenin-mediated quality traits. Consistent with these findings, previous genome-wide association studies have shown that core dough quality traits are governed by stable genetic networks and are less affected by agronomic conditions. A recent multi-model GWAS confirmed that grain hardness QTLs are highly stable across environments, with 13 QTNs consistently detected in both tested environments, further validating the environmental robustness of quality-related genetic loci (Eltaher et al., 2021; Jin et al., 2023; Bhadana et al., 2026).

In contrast, grain protein content exhibited strong environmental responsiveness, with site contributing 22.35% and year contributing 22.74% (**Table 4**). The comparable magnitude of site and year contributions indicates that spatial ecological heterogeneity and interannual climate variability exert equivalent constraints on protein formation in the Ili River Valley—a pattern characteristic of arid irrigation systems where both site-specific soil–water–nutrient conditions and seasonal climate fluctuations jointly limit nitrogen metabolism. Protein accumulation involves nitrogen uptake, assimilation, and grain translocation, processes that are highly sensitive to water and nutrient availability (El Baouchi et al., 2024). The high environmental contribution observed here is consistent with previous studies showing that genotype, environment, and crop management jointly determine wheat quality traits, with environment being the dominant factor for protein variation across diverse agroecosystems (Studnicki et al., 2016).

Notably, wet gluten content exhibited a hybrid G × E pattern distinct from both rheological traits and protein content, with genotypic contribution of 44.25%, site contribution of 22.25%, and management factors (water 13.28% and nitrogen 7.54%) also making substantial contributions (**Table 4**). This intermediate pattern indicates that wet gluten formation is co-regulated by both genetic factors and field management, making it a trait particularly responsive to water–nitrogen coupling regulation. This intermediate positioning of wet gluten as a bridging trait underscores its utility as a key indicator for evaluating the efficacy of water–nitrogen coupling strategies, as it integrates both genetic potential and management inputs (Bhadana et al., 2026). Recent studies have confirmed that wet gluten content is significantly affected by both irrigation and nitrogen management, with high-frequency drip fertigation increasing wet gluten by 4.5%–22.1% (Hao et al., 2023), and that wet gluten responds differently from protein content to water–nitrogen interactions due to its dual dependence on both protein quantity and quality composition (Fan et al., 2025).

Importantly, the environment-dependent nature of protein content does not preclude the existence of genotypes with superior environmental buffering capacity. Among the eight tested cultivars, Neimai 17 exhibited the lowest AMMI stability value (ASV) for protein content (0.21) and the lowest mean ASV across all quality traits (0.38), ranking first in overall stability (**Table 6**). Rather than contradicting the high environmental contribution to protein variation, this stability highlights the practical value of identifying genotypes with strong nitrogen utilization homeostasis that can effectively buffer external resource fluctuations (Sheta et al., 2024). Recent studies have shown that moderate water limitation during grain filling can enhance nitrogen remobilization from source to sink organs through cytokinin redistribution, improving nitrogen harvest index and nitrogen use efficiency (Liu et al., 2026). This mechanism may partially explain why genotypes like Neimai 17 maintain relatively stable protein content under water fluctuations, suggesting that superior nitrogen homeostasis in these cultivars may involve cytokinin-mediated regulatory pathways. Such genotypes are precisely the material basis needed for quality improvement in arid regions, where environmental variability is unavoidable and agronomic regulation alone is insufficient to guarantee stable quality performance.

### Regional specificity of water–nitrogen coupling regulation on wheat quality

This study verified that water–nitrogen coupling balance modulates spring wheat quality formation in arid oasis irrigation areas, with regulation effects that differ across quality traits and show regional specificity compared with humid wheat-growing regions.

Regarding grain protein content, although the overall water and nitrogen effects in the ANOVA were small (0.18% and 1.22%, respectively; **Table 4**), the treatment-level comparison revealed a clear pattern at the N240 level: W450 produced the highest protein content (13.76%), significantly exceeding both W300 (13.59%) and W600 (13.57%) (**Table 3**). This non-linear response indicates that excessive irrigation at moderate nitrogen input does not further enhance but instead slightly suppresses protein accumulation, consistent with the dilution effect of excess water on grain nitrogen concentration. Recent research on winter wheat has revealed the underlying mechanism of this dilution effect: protein and starch exhibit opposite responses to irrigation—protein content is negatively affected by excessive irrigation due to carbohydrate dilution, while starch accumulation benefits from higher water availability, resulting in a decreased protein-to-starch ratio under sufficient irrigation (Zib et al., 2025). In contrast, at the N0 level, W600 produced the highest protein (13.73%), which was likely because moderate water availability under nitrogen-deficient conditions helps maintain the metabolic activity required for protein degradation in source organs and amino acid translocation to grains (Liu et al., 2026). This divergent response across nitrogen levels illustrates the interactive nature of water–nitrogen coupling on protein formation.

Wet gluten content showed the most pronounced response to water–nitrogen treatments among all quality traits, with water and nitrogen contributions of 13.28% and 7.54%, respectively (**Table 4**), both highly significant (*P* < 0.001). The N240 + W450 treatment achieved the highest wet gluten content (32.66%), while the N240 + W600 treatment dropped significantly to 31.09% (**Table 3**), a reduction of 1.57%. This substantial decline confirms that excessive irrigation directly degrades wet gluten quality under optimal nitrogen supply, consistent with findings that irrigation level significantly affects wet gluten content (Hao et al., 2023; Fan et al., 2025).

The regional specificity of these findings deserves emphasis. In traditional humid and semi-humid wheat regions, nitrogen input is the primary regulatory measure for protein quality improvement. In this study, however, the nitrogen use efficiency for protein was remarkably low: at W450, increasing nitrogen from N0 to N240 raised the protein content by only 0.60% over 240 kg·hm^-2^, equivalent to approximately 0.08% per 30 kg·hm^-2^ nitrogen increment. At W300, the same nitrogen increment yielded only 0.24% (0.03 per 30 kg·hm^-2^), while at W600, the N0-to-N240 transition actually decreased protein by 0.16% (**Table 3**). These values are far below the typical nitrogen efficiency reported in humid regions (Fuertes-Mendizábal et al., 2010), indicating that water scarcity and supply fluctuations severely constrain nitrogen effectiveness in arid environments. This is consistent with recent studies in arid irrigation systems demonstrating that water deficit significantly limits nitrogen uptake and translocation efficiency and that the interaction between irrigation level and nitrogen rate is the primary determinant of grain protein variation rather than nitrogen rate alone (Huang et al., 2024; Zong et al., 2026). Studies on drip-irrigated spring wheat in Xinjiang have confirmed that mild drought during the tillering stage followed by re-watering can promote dry matter accumulation and increase grain weight (Ma et al., 2025). However, the present study focuses on quality responses, and the optimal water–nitrogen combination for quality traits may differ from that for yield, suggesting that quality-oriented and yield-oriented management strategies may require distinct irrigation and nitrogen regimes in this region.

It should also be noted that the response of different quality traits to excessive irrigation was not uniformly negative. At the N240 level, W600 produced the highest extensograph area (105.78 cm²) and resistance to extension (444.38 EU), exceeding the N240 + W450 treatment (101.42 cm² and 412.59 EU, respectively; **Table 3**). This divergence—where excessive irrigation simultaneously reduces protein and wet gluten but enhances dough rheological strength—reflects the trait-specific G × E differentiation discussed in Section 'Trait-specific genetic and environmental dominance governing wheat quality'. Rheological traits, being predominantly genotype-controlled, are less responsive to water fluctuations in directionality and may benefit from enhanced starch hydration and swelling under higher water supply, which strengthens the starch–protein interaction network and increases dough resistance (Han et al., 2025). Consistent with this, recent research on winter wheat has demonstrated that starch accumulation benefits from adequate irrigation, which enhances the starch–protein interaction network and improves dough rheological properties (Zib et al., 2025). A recent study on wheat dough quality formation mechanisms revealed that water availability modulates the ratio of disulfide bonds to hydrogen bonds in the gluten network, where higher water content promotes hydrogen bond formation and enhances dough extensibility, while moderate drought promotes disulfide bond-mediated protein pre-aggregation and increases dough strength (Yang et al., 2025). This provides a mechanistic basis for understanding the differential responses of protein quantity and dough rheological properties to irrigation regimes observed in this study.

The significant site × water interaction for wet gluten (2.03%, *P* < 0.01) and resistance to extension (1.99%, *P* < 0.001) further indicates that water regulation effects differ across ecological sites (**Table 4**), reinforcing the need for site-specific water–nitrogen management strategies rather than uniform recommendations.

### Optimization and applicability of integrated AMMI–GGE evaluation system

Single statistical models have obvious limitations in multi-environment wheat variety evaluation, and the defects of independent AMMI or GGE application are empirically reflected in the data analysis of this study rather than limited to general theoretical consensus.

The AMMI model can quantify variety stability through ASV, but it cannot distinguish the specific adaptive environment of superior varieties. In this study, AMMI analysis showed that Neimai 17 had the lowest protein ASV (0.21) and the lowest mean ASV (0.38) across all traits, ranking first in stability (**Table 6**). However, AMMI alone cannot reveal whether this stability is universal across all environments or concentrated in specific sites. The AMMI IPCA analysis for protein content showed that the first two principal components explained 83.87% of the interaction variation (IPCA1: 58.87%, IPCA2: 25.00%; **Table 5**), indicating a substantial G × E interaction structure that requires further spatial interpretation. This limitation of AMMI—providing quantitative stability without environmental specificity—has been recognized in recent multi-method comparison studies. Shahin et al. (2024) noted that while AMMI provides phenotypic stability and genetic divergence information, it cannot delineate mega-environments or identify the most representative test environments, a function uniquely served by GGE biplot analysis.

GGE biplot analysis complemented this by providing visual variety–environment interaction patterns. For extensograph area, the GGE biplot captured 98.00% of the total variation with the first two principal components (PC1: 96.96%, PC2: 1.04%; **Table 7**), reflecting the overwhelming genotypic dominance of this trait. For protein content, the cumulative interpretation rate was 88.52% (PC1: 80.23%, PC2: 8.29%), with the higher PC2 contribution suggesting more complex variety–environment interaction patterns. However, GGE alone cannot quantitatively decompose the relative contributions of genotypic, environmental, and interactive effects—information provided by the ANOVA and AMMI stability parameters. Furthermore, relying solely on GGE without AMMI’s quantitative stability decomposition may identify a variety as being well-adapted to a specific environment based on visual clustering yet overlook substantial within-environment performance variability that undermines practical reliability—a risk particularly relevant when the number of test environments is limited and interaction patterns are complex. Recent multi-method evaluation studies have increasingly recognized that integrating AMMI stability parameters with GGE environmental classification is more effective than using either method alone, as this combination provides both quantitative stability and qualitative adaptability information simultaneously (Gerema et al., 2024).

The integrated AMMI–GGE framework adopted in this study realizes complementary advantages: AMMI provides accurate quantitative stability parameters, and GGE achieves intuitive environmental adaptability positioning. Based on this multi-index system, Neimai 17 was accurately screened as the top-ranked cultivar, ranking first in both AMMI mean ASV (0.38; **Table 6**) and GGE comprehensive score (13.61; **Table 8**). The concordance between two independent evaluation methods strengthens the reliability of this selection. This result proves that multi-model fusion can effectively avoid the one-sided evaluation deviation of single models and single traits, providing a more scientific and reliable method for regional wheat variety screening and layout.

## Conclusions

Based on 2-year (2022–2023) field trials conducted across three typical ecological regions (Gongliu, Tekes, Zhaosu) of the Ili River Valley, this study systematically analyzed the effects of genotype (G), environment (E), and their interaction (G × E) on six core processing quality traits of spring wheat using combined AMMI, GGE biplot, and multivariate evaluation approaches. The results revealed that spring wheat quality was synergistically regulated by G, E, and G × E, with strong trait-specific G × E patterns: dough rheological traits (extensograph area and resistance to extension) were genotype-dominated (79.02% and 72.71%), while grain protein content was environment-dominated (45.09%), reflecting fundamental differences in genetic stability and physiological plasticity among quality traits. Among the three regions, Gongliu exhibited the best comprehensive quality performance, indicating its ecological suitability for high-quality wheat production. Water–nitrogen coupling significantly modulated quality formation, and N240 + W450 was identified as the optimal combination, balancing high quality and nitrogen use efficiency under arid irrigation conditions. Variety screening showed that Neimai 17 had the lowest mean ASV (0.38) and the highest comprehensive score, demonstrating excellent environmental buffering capacity and stable quality performance across variable water–nitrogen and ecological conditions. The integrated AMMI–GGE framework effectively quantified stability and visualized adaptability, providing a robust multi-model strategy for evaluating spring wheat quality and stability in multi-environment trials. These findings enhance the theoretical understanding of trait-specific G × E regulation and water–nitrogen coupling mechanisms in arid spring wheat and provide practical guidance for variety deployment, precise water–nitrogen management, and high-quality, efficient wheat production in the Ili River Valley and similar northwest arid regions of China.

## Data Availability

The original contributions presented in the study are included in the article/**Supplementary Material**. Further inquiries can be directed to the corresponding author.

## Supplementary material

Supplementary Table 1 Basic information of tested wheat varieties.

Supplementary Table 2 Classification standards of wheat with four quality grades.
