# Supplementary material for: Evaluation of multi-environment adaptability of flour quality traits in spring wheat varieties and screening of high-quality genotypes using AMMI and GGE models
Source: Front Plant Sci. 2026 Jul 10;17:1874719. doi: 10.3389/fpls.2026.1874719 (PMC13397585; doi:10.3389/fpls.2026.1874719)
Supplement: Supplementary Table 1 — Basic information of tested wheat varieties. [file Table1.pdf]

Table S1. Basic information of tested wheat varieties

Variety	Gluten strength type	Core characteristics	Main promotion areas	Seed source
Xinchun 37	Strong gluten	Spring growth period of 94 to 99 days moderate plant height of 85 to 90 centimeters lodging resistance stable flour water absorption medium stability time suitable for popular wheat products	Spring wheat areas of the whole Xinjiang eastern Qinghai	Institute of Food Crops Xinjiang Academy of Agricultural Sciences
Xinchun 44	Strong gluten	Spring growth period of 97 to 102 days high-quality strong gluten type carrying high-quality subunits of Glu-1 allele high gluten strength high sedimentation value suitable for bread processing	High-quality wheat producing areas such as Yili and Changji in Xinjiang	Institute of Food Crops Xinjiang Academy of Agricultural Sciences
Neimai 17	Medium strong gluten	Spring growth period of 100 to 105 days stripe rust resistance large spike and heavy grain high protein content above 16 percent high wet gluten content strong resistance to extension	Spring wheat areas such as Yili in Xinjiang and Yinchuan in Ningxia	Institute of Crops Ningxia Academy of Agriculture and Forestry Sciences
Hechun 137	Medium strong gluten	Spring growth period of 95 to 100 days compact plant type strong lodging resistance plump grains high flour extraction rate medium gluten elasticity	Spring wheat areas such as Yili and Tacheng in Xinjiang	Institute of Food Crops Xinjiang Academy of Agricultural Sciences
Liangchun 1201	Medium gluten	Spring growth period of 98 to 103 days strong tillering ability outstanding drought resistance hard grains medium protein content suitable for steamed bun and noodle processing	Northern Xinjiang spring wheat area Hexi Corridor in Gansu	Institute of Crops Gansu Academy of Agricultural Sciences
Ningchun 38	Medium gluten	Spring growth period of 96 to 101 days strong stress resistance cold and barren tolerance uniform grains high flour whiteness excellent cooking quality	Spring wheat areas in Ningxia Gansu and northern Xinjiang	Institute of Crops Ningxia Academy of Agriculture and Forestry Sciences
Xinchun 48	Medium weak gluten	Spring growth period of 93 to 98 days good yield potential high thousand kernel weight of 45 to 50 grams wide flour processing adaptability high sensory score of steamed buns	Northern Xinjiang spring wheat area western Inner Mongolia	Institute of Food Crops Xinjiang Academy of Agricultural Sciences
Nanmai 660	Weak gluten	Spring growth period of 92 to 96 days early maturity wide adaptability soft grains low protein content high starch content suitable for pastry processing	Southern Xinjiang spring wheat area Sichuan Basin spring wheat area	Institute of Crops Sichuan Academy of Agricultural Sciences
