# Supplementary material for: Evaluation of multi-environment adaptability of flour quality traits in spring wheat varieties and screening of high-quality genotypes using AMMI and GGE models
Source: Front Plant Sci. 2026 Jul 10;17:1874719. doi: 10.3389/fpls.2026.1874719 (PMC13397585; doi:10.3389/fpls.2026.1874719)
Supplement: Supplementary Table 2 — Classification standards of wheat with four quality grades. [file Table2.pdf]

Table S2. Classification standards of wheat with four quality grades

Classification	Protein (%)	Wet gluten content (%)	Water absorption (%)	Stability time (min)	Resistance to extension (BU)	Extensograph area (cm^2)
Strong gluten	≥14	≥30.5	≥60	≥10	≥450	≥100
Medium strong gluten	≥13	≥28.5	≥58	≥7	≥350	≥80
Medium gluten	≥12	≥24	≥55	≥3	≥200	≥50
Weak gluten	<12	<24	<55	<3	—	—
